# Gut microbiota and Parkinson’s disease: exploring pathogenesis and potential therapeutic strategies from a gut-brain axis perspective

**DOI:** 10.1016/j.isci.2025.114185

**Published:** 2025-12-04

**Authors:** Xiayu Jin, Jirui Wei, Xudong Min, Yiqi Fan, Zhao Yuan, Zhuolin Du, Zequn Su, Tianrong Xun, Qingyao Du, Tiecheng Liang, Xiaozheng He, Waijiao Tang

**Affiliations:** 1 Neurosurgery Center, Department of Functional Neurosurgery, National Key Clinical Specialty, Engineering Research Center of Diagnostic and Therapeutic Technology and Devices for Cerebrovascular Diseases in Ministry of Education, Guangdong Provincial Key Laboratory on Brain Function Repair and Regeneration, Zhujiang Hospital Institute for Brain Science and Intelligence, Zhujiang Hospital, Southern Medical University, Guangzhou 510282, China; 2 Drug Clinical Trial Institution, Department of Pharmacy, Zhujiang Hospital, Southern Medical University, Guangzhou 510282, China; 3 Department of Pharmacy, Shenzhen Hospital, Southern Medical University, Shenzhen, China; 4 The National Key Clinic Specialty, The Engineering Technology Research Center of Education Ministry of China, Guangdong Provincial Key Laboratory on Brain Function Repair and Regeneration, Department of Neurosurgery, Zhujiang Hospital, Southern Medical University, Guangzhou 510282, China

**Keywords:** Neuroscience, Microbiology

## Abstract

Parkinson’s disease (PD) is a prevalent neurodegenerative disorder with a global prevalence exceeding 1‰, posing a significant public health challenge. Although the pathogenesis of PD is not yet fully elucidated, accumulating evidence suggests that it results from the interplay between genetic and environmental factors, highlighting its multifactorial nature. With advances in translational medicine, the gut has emerged as a critical participant in PD onset and progression. This review systematically summarizes the role of the gut in PD, particularly emphasizing potential mechanisms involving neuroinflammation in the central nervous system (CNS), pathological aggregation of α-synuclein (α-syn), and mitochondrial dysfunction. Furthermore, gut-targeted therapeutic strategies for PD are discussed, including fecal microbiota transplantation (FMT), gut-directed anti-inflammatory therapies, supplementation with gut microbiota-derived metabolites such as short-chain fatty acids (SCFAs), and interventions targeting α-syn aggregation. A deeper understanding of these mechanisms not only advances the pathological knowledge of PD but also provides theoretical foundations for the early diagnosis and innovative treatment of the disease.

## Graphical abstract

**Figure:**
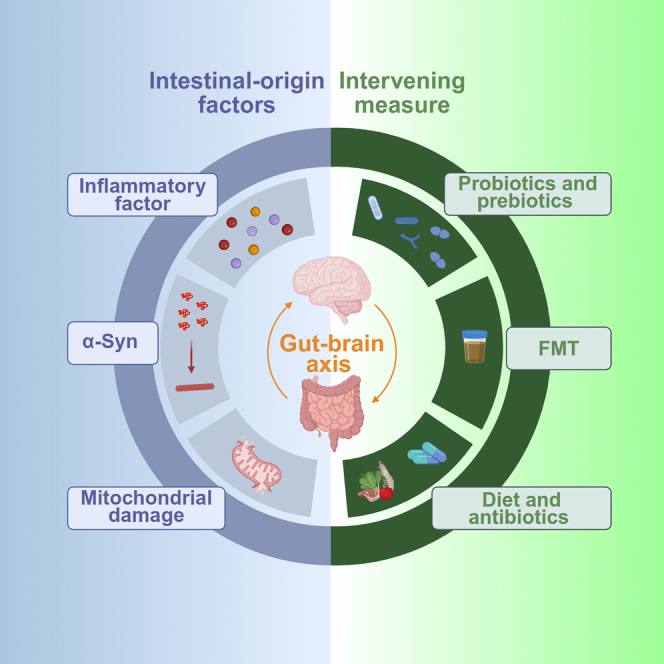

## Introduction

PD is the second most common neurodegenerative disorder globally,^1^characterized primarily by the progressive degeneration of dopaminergic neurons in the substantia nigra pars compacta (SNpc) and abnormal accumulation of α-syn forming Lewy bodies (LBs).^2^ Beyond hallmark motor symptoms such as tremor, rigidity, bradykinesia, and postural instability,^3^ Patients with PD also present with non-motor symptoms, notably constipation, which typically precede motor manifestations.^4^ This suggests that peripheral systems, especially the gastrointestinal (GI) tract, play a crucial role in PD pathogenesis.^5^

According to the well-established Braak hypothesis, pathological changes in PD might originate in the gut and subsequently spread to the CNS through the vagus nerve (VN).^6^^,^^7^ The recent advances in understanding the gut-brain axis—a complex bidirectional communication network involving neural, endocrine, immune, and metabolic pathways—have further supported this hypothesis. This axis integrates the CNS, autonomic nervous system (ANS), enteric nervous system (ENS), hypothalamic-pituitary-adrenal (HPA) axis, and gut microbiota.^8^ Communication along this axis occurs directly through the VN and autonomic pathways or indirectly via the ENS.^9^ Additionally, gut microbiota-derived metabolites such as SCFAs, neurotransmitters (dopamine, serotonin, γ-aminobutyric acid), and hormones (e.g., corticotropin-releasing hormone) mediate gut-brain interactions.^10^^,^^11^^,^^12^

The gut, often termed the “second brain,” houses the largest microbial community interacting with the host, and the role of gut microbiota in neurological diseases has become a significant research focus in recent years. Numerous studies have reported distinct changes in the gut microbiota composition of patients with PD compared to healthy individuals. For instance, increased abundance of Bifidobacterium, Lactobacillus, Enterococcus, and Akkermansia, and decreased levels of Blautia, Coprococcus, and Prevotellaceae have been observed consistently in PD cohorts.^13^^,^^14^^,^^15^ Importantly, similar patterns of microbiota dysbiosis have been confirmed across various animal models of PD, including SNCA-A53T transgenic mice and neurotoxin-induced models (e.g., MPTP-treated mice).^16^ These cross-species findings indicate that gut microbiota dysbiosis may influence PD pathology by modulating α-syn aggregation, neuroinflammation, and metabolic homeostasis, thereby supporting microbiota-targeted therapeutic interventions.

**Figure 1:**
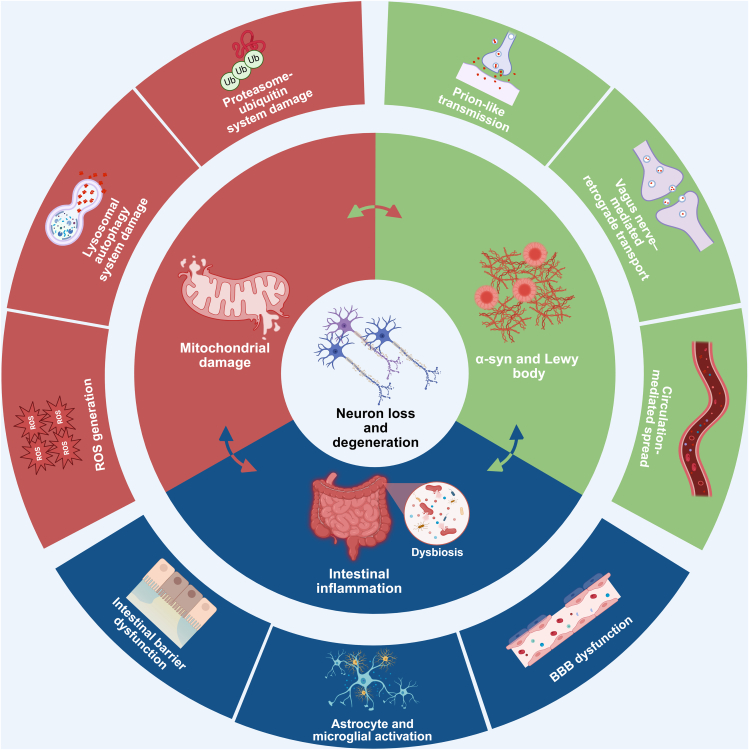
Key gut-brain axis mechanisms contributing to neurodegeneration in Parkinson’s disease The central circle depicts neuron loss and degeneration; the middle ring summarizes three major pathogenic axes—intestinal inflammation, mitochondrial damage, and α-syn and Lewy body pathology—while the outer ring illustrates representative mechanisms associated with each axis.

**Figure 2:**
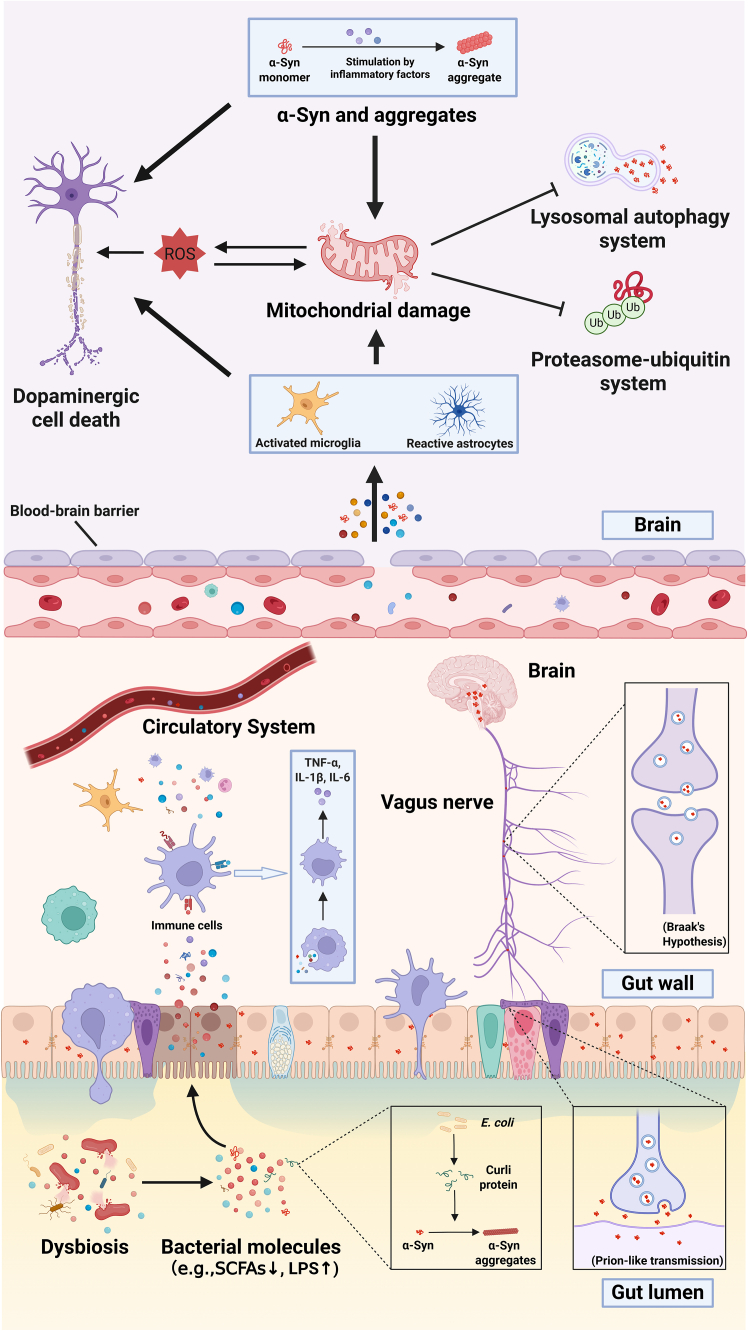
Mechanistic schematic of the microbiota-gut-brain axis in Parkinson’s disease Gut dysbiosis, with reduced beneficial (e.g., SCFA-producing) microbes and increased detrimental metabolites, disrupts intestinal barrier integrity, enabling bacterial products to penetrate the mucosa, activate immune cells, and induce pro-inflammatory cytokines that reach the CNS via the circulation, thereby activating glial cells and damaging dopaminergic fibers. In parallel, the inflammatory intestinal milieu promotes epithelial release of misfolded α-syn, whose aggregates propagate retrogradely to the CNS via the vagus nerve, while within the brain, persistent neuroinflammation and toxic protein aggregates drive mitochondrial dysfunction and impaired protein degradation pathways, ultimately exacerbating α-syn pathology, dopaminergic neuron loss, and circuit disruption in PD.

Current evidence suggests two principal mechanisms through which gut microbiota influence brain functions. Firstly, gut bacteria directly produce neurotransmitters such as acetylcholine, dopamine, norepinephrine, and serotonin, thereby affecting brain activity.^17^^,^^18^ Secondly, microbiota-derived metabolites, notably SCFAs, mediate the bidirectional gut-brain axis communication.^12^^,^^19^ In summary, perturbations of the gut microbiota and alterations in GI function may occur in the early stages of PD, suggesting a potential role for gut dysfunction in both the initiation and progression of the disorder. To facilitate a more comprehensive understanding of the multifaceted contributions of the gut ecosystem to PD pathophysiology, we present an integrated mechanistic illustration (Figure 1). This schematic outlines the pathological interactions between the gut and the CNS through the microbiota-gut-brain axis, encompassing key processes such as inflammatory signaling cascades, pathological aggregation of α-syn, and mitochondrial dysfunction. Building on this framework, Figure 2 further details how gut dysbiosis, barrier disruption, microbial products, and misfolded α-syn cooperate along the microbiota-gut-brain axis to drive dopaminergic neurodegeneration. The present review centers on three core mechanisms underlying PD pathogenesis—neuroinflammation, aberrant α-syn pathology, and impaired mitochondrial function—and explores how gut-derived factors intersect with these processes. Furthermore, therapeutic strategies targeting the gut microbiota and intestinal milieu are discussed, offering a conceptual basis for future innovation in PD treatment paradigms.

### Gut-mediated central nervous system inflammation and its mechanisms in Parkinson’s disease

For a long time, the CNS was considered to be an immune-privileged site. However, since the 20th century, this view has gradually shifted toward the understanding that peripheral immune systems participate in a complex brain immune network.^20^ It is now widely accepted that adult neurogenesis, or the lifelong formation of new neurons, is supported by adaptive immune cells.^21^ Dysregulated immune responses, particularly neuroinflammation, are implicated in neuronal injury and are associated with multiple CNS disorders.^22^ In the 1980s, studies observed activation of microglia and elevated levels of inflammatory markers in the brains of patients with PD,^23^and these inflammatory factors were found to induce neuronal necrosis and apoptosis.^24^ Furthermore, the abundance of gut microbiota that negatively correlates with inflammation, such as Coprococcus, Roseburia, and Blautia, is significantly reduced in patients with PD.^25^ Similar changes have also been observed in patients with Alzheimer’s disease (AD) and autism spectrum disorder (ASD).^26^^,^^27^ These findings suggest that intestinal inflammation might contribute to the development and progression of CNS diseases through the gut-brain axis. The relationship between the gut and CNS inflammation is frequent and complex. Broadly speaking, the gut can influence CNS inflammation through three main mechanisms: 1) gut barrier integrity, 2) immune response, and 3) gut microbiota. These mechanisms will be explored in detail to elucidate the connections between the gut and CNS inflammation.

#### Relationship between impaired gut barrier function and central nervous system inflammation

The gut barrier is primarily composed of the vascular barrier, epithelial barrier, and the mucus layer within the gut, with the luminal side in direct contact with the intestinal contents and the tissue side closely linked to gut neurons and glial cells.^28^ The gut barrier performs dual functions in preventing the pathogenic infiltration of microorganisms and toxic substances while simultaneously mediating selective absorption of aqueous solutions and nutrients. Studies have shown that patients with PD often exhibit impaired gut barrier function, with a reduction in the expression of tight junction proteins such as Occludin, Claudin-1, and Zonula Occludens-1, leading to increased intestinal permeability.^29^ Once the gut barrier is compromised, bacteria, lipopolysaccharide (LPS), and other toxins are more likely to enter the systemic circulation, triggering a systemic immune response.^30^ Furthermore, these harmful substances may cross the blood-brain barrier, potentially influencing CNS function either directly or indirectly.^31^

#### Gut immune dysregulation induces neuroinflammation through toll-like receptors pathways

Pattern recognition receptors (PRRs), as the first line of defense in the innate immune system, primarily recognize pathogen-associated molecular patterns (PAMPs) and damage-associated molecular patterns (DAMPs). Among these, TLRs are widely recognized for their role in detecting both PAMPs and DAMPs, activating downstream immune responses.^32^ TLRs are expressed not only on peripheral immune cells but also on neurons and glial cells within the CNS.^33^ Pathogen products, such as bacterial endotoxins such as LPS, entering the peripheral circulation can activate TLRs on immune cells, particularly TLR4, initiating the MyD88-dependent signaling pathway and leading to the activation of nuclear factor kappa B (NF-κB).^34^^,^^35^ NF-κB, as a critical transcriptional regulator, promotes the expression of various pro-inflammatory cytokines, including tumor necrosis factor-alpha (TNF-α), interleukin-1β (IL-1β), and interleukin-6 (IL-6).^36^ These inflammatory cytokines not only provoke peripheral inflammation but can also cross the blood-brain barrier (BBB), activating microglia within the CNS, increasing the secretion of pro-inflammatory factors, and exacerbating the inflammatory cascade, ultimately leading to neuroinflammation and neuronal damage. This creates a state of chronic inflammation within the CNS.^37^ Such chronic inflammation further increases BBB permeability, allowing more peripheral inflammatory cytokines and toxic substances to infiltrate the CNS, thereby intensifying neuronal damage.^38^ Additionally, chronic neuroinflammation can activate astrocytes, impairing their neuroprotective and metabolic support functions for neurons.^39^

Recent studies suggest that TLR2-mediated inflammatory pathways also play a significant role in the pathogenesis of PD.^40^ One study demonstrated that TLR2 knockout mice exhibited reduced motor dysfunction and alleviated degeneration of dopaminergic neurons in the nigrostriatal pathway following 1-methyl-4-phenyl-1,2,3,6-tetrahydropyridine (MPTP) treatment. This improvement was closely associated with the inhibition of the TLR2/MyD88/TRAF6/NF-κB signaling pathway, reduced abnormal α-syn aggregation, and decreased astrocyte activation and neuroinflammation.^41^ Moreover, TLR4 knockout mice showed significant reductions in gut and neuroinflammation as well as improved motor function following rotenone treatment, further underscoring the role of TLR4 in both gut and CNS inflammation.^42^

On the other hand, gut inflammation can also trigger the activation of specific T cell subsets, such as T helper 17 (Th17) cells.^43^ Studies have shown that IL-17, a cytokine secreted by Th17 cells, plays a critical role in neuroinflammation in PD.^44^^,^^45^ IL-17 increases BBB permeability, promoting the migration of peripheral immune cells into the CNS, thus exacerbating neuroinflammation and neuronal damage.^46^ Recent research has found that transferring fecal microbiota from patients with PD to wild-type C57BL/6 mice induced immune dysregulation, including Th17 activation, which further affected the brain and led to PD-like pathological changes.^43^

In summary, the damage to gut barrier function and the abnormal activation of inflammatory pathways can intensify inflammatory responses in both peripheral systems and the CNS, playing a pivotal role in the pathogenesis and progression of PD. Protecting gut barrier integrity and inhibiting excessive inflammation could be potential therapeutic strategies for PD. Additionally, the VN, a key component of the gut-brain axis, also plays an essential role in immune signal transmission.^47^ When the gut microbiota is dysregulated, the VN transmits relevant immune signals to the brain, further activating central immune cells and inducing or worsening neuroinflammation.^48^ Moreover, a bidirectional neuro-immune regulatory mechanism exists between the VN and gut microbiota, which collectively influences immune homeostasis in the CNS.^49^ Therefore, gut dysbiosis and inflammation may contribute to neuroinflammation through the gut-brain axis, potentially triggering or exacerbating PD. While there is still debate on whether this mechanism serves as an initiating factor in PD pathogenesis, its critical role in the progression of chronic inflammation during the disease course is increasingly acknowledged.

#### Gut microbial community and microbial metabolites as drivers of Parkinson’s disease onset and progression

In recent years, an increasing number of studies have demonstrated a close relationship between gut microbiota dysbiosis and the onset and progression of various neurological diseases.^50^^,^^51^ The gut microbiota plays an essential role in regulating the bidirectional communication network between the gut and brain, referred to as the “microbiota-gut-brain axis.”^52^ In a healthy state, the gut microbiota exhibits high diversity and stability, maintaining a dynamic symbiotic relationship with the host.^53^ However, an imbalance in the gut microbiota can significantly affect the physiological functions of the brain, cognitive abilities, and behavioral patterns, leading to growing interest in research surrounding the “microbiota-gut-brain axis.”^54^^,^^55^ Current evidence from several studies suggests that various neurological and psychiatric diseases, including AD, multiple sclerosis (MS), stroke, and PD, as well as anxiety and depression, are closely linked to gut microbiota dysbiosis.^56^^,^^57^^,^^58^^,^^59^ Recent metagenome-wide association studies have reported that alterations in the gut microbiome are associated with PD risk; however, causality remains to be established.^60^^,^^61^

Gut microbiota, as a key regulator of the gut-brain axis, is a key regulator of gut-brain communication and contributes to PD-related neuroinflammation. Numerous studies have shown that patients with PD exhibit significant gut microbiota imbalance, which may be closely related to disease progression and the onset of GI symptoms at specific stages of the disease.^53^^,^^62^ Recent large-sample metagenomic studies and multicenter meta-analyses indicate that the gut microbiota of patients with Parkinson’s disease (PD) exhibits a phylogenetic signature characterized by reduced abundances of butyrate-producing taxa and increased abundances of conditionally pathogenic bacteria. Genera typically depleted include the butyrate producers *Faecalibacterium*, *Roseburia*, *Coprococcus*, and *Prevotella*, whereas enrichments are observed in Enterobacteriaceae, *Akkermansia*, *Clostridium*, and certain *Desulfovibrio*.^63^ This dysbiosis corresponds to pathological features in PD, including reduced fecal SCFAs, compromised intestinal barrier integrity, and activation of inflammatory pathways. For example, the abundance of Prevotella, a genus with recognized anti-inflammatory properties, is significantly reduced in the gut microbiota of patients with PD, and this reduction may exacerbate inflammatory responses.^29^^,^^64^ Additionally, an increase in Enterobacteriaceae, a family that includes pathogenic bacteria such as Escherichia coli, can lead to the production of endotoxins, which further promote inflammation. A rise in Clostridium species may also release endotoxins, further driving inflammation.^42^^,^^65^ Munoz-Pinto and colleagues found that transferring fecal microbiota from patients with PD into the gut of wild-type male C57BL/6 mice resulted in changes to the ileal mucosal microbiota composition, disrupted Th17 cell immune homeostasis, and triggered intestinal inflammation that subsequently spread to the brain, inducing PD-like pathophysiological changes.^43^ Similar studies have demonstrated that FMT from patients with PD significantly aggravated the inflammatory response and neurodegeneration in A53T transgenic mice.^66^ Moreover, in an aged mouse model of PD induced by rotenone, oral administration of rotenone led to changes in gut microbiota composition, activation of systemic inflammatory responses, inhibition of PPARγ signaling, and increased neuroinflammation and ferroptosis, with chemokine CXCL1 playing an important role in these pathological processes.^67^ Table 1 summarizes other studies on gut microbiota alterations in patients with PD.

**Table 1:** Alterations of gut microbiota in PD and associated mechanisms

Microbial taxa	Alteration	Proposed mechanisms	Reference
Firmicutes	Decreased	Fermentation of dietary fiber producing SCFAs,^68^ which possess anti-inflammatory and immunomodulatory properties.^69^	Rada-Iglesias et al. (2007)^70^
Bacteroidetes	Decreased	Similar functions to Firmicutes, involving the production of SCFAs with anti-inflammatory and immunomodulatory activities.	Rada-Iglesias et al. (2007)^70^
Ralstonia	Increased	Production of pro-inflammatory mediators, such as LPS.	Keshavarzian et al.(2015)^25^
Faecalibacterium	Decreased	A major butyrate producer with anti-inflammatory properties.	Keshavarzian et al.(2015)^25^
Blautia	Decreased	Key producers of SCFAs, particularly butyrate.	Keshavarzian et al.(2015)^25^
Coprococcus	Decreased	Important SCFA-producing bacteria, particularly butyrate.	Keshavarzian et al.(2015)^25^
Roseburia	Decreased	Prominent SCFA-producing genus, notably involved in butyrate synthesis.	Keshavarzian et al.(2015)^25^
Desulfovibrio	Increased	Sulfate-reducing bacteria commonly found in gut induce inflammatory cytokines (e.g., TNF-α, iNOS) via TLR2-dependent PI3K/Akt signaling pathways.	Singh et al. (2024)^71^
Citrobacter rodentium	Increased	Elevation of inflammatory markers such as TLR4 and NF-κB p65 in the colon and striatum of PD mouse models aggravates neuroinflammation.	He et al. (2024)^72^
Akkermansia muciniphila	Decreased	Reduction of colonic inflammation, amelioration of neuroinflammation in the striatum and hippocampus, improvement of α-syn aggregation, and increased fecal iso-valerate levels in PD mouse models.	Qiao et al. (2024)^73^

SCFAs, primarily produced through the colonic microbial fermentation of dietary fibers, mainly include acetate (∼60%), propionate (∼25%), and butyrate (∼15%).^58^^,^^74^^,^^75^ Under physiological conditions, SCFAs play critical roles in maintaining gut barrier integrity, regulating host immune responses, and serving as energy substrates.^30^^,^^75^ Acetate and propionate are predominantly generated by bacteria within the Bacteroidetes phylum, while butyrate is mainly produced by Firmicutes.^76^ Although butyrate represents a smaller fraction of SCFAs, it is typically considered the most biologically active.^77^

Research has indicated that butyrate significantly improves intestinal dysfunction and motor deficits in PD animal models by inhibiting the TLR4/MyD88/NF-κB inflammatory pathway, thereby blocking the transmission of inflammatory signals along the gut-brain axis and providing neuroprotective effects.^78^ Furthermore, butyrate can inhibit microglial activation through modulating the renin-angiotensin system (RAS)-NF-κB signaling pathway, reducing neuroinflammation and dopaminergic neuronal apoptosis in mice—a pathway alteration also observed in patients with PD.^79^ Additionally, butyrate exerts neuroprotective effects by suppressing the JAK2/STAT3 signaling pathway in MPTP or MPP(+) treated animal and cellular models of PD.^80^ Besides its anti-inflammatory properties, butyrate has been shown to decrease blood-brain barrier permeability and improve PD-related depressive symptoms.^81^

Collectively, SCFAs exert their anti-inflammatory effects mainly by activating G protein-coupled receptors (such as GPR41 and GPR43) and inhibiting histone deacetylase (HDAC) activity, thus modulating gene expression.^82^^,^^83^^,^^84^ Reduced levels of SCFAs in the gut of patients with PD may exacerbate local intestinal inflammation.^70^ A deficiency in SCFA-mediated anti-inflammatory function can lead to the abnormal activation of the NF-κB signaling pathway and consequently induce the excessive expression of pro-inflammatory cytokines.^68^ Additionally, reduced SCFA levels can compromise gut barrier integrity, increasing the translocation of bacteria and their metabolites into systemic circulation, triggering systemic inflammation.^12^ Therefore, SCFAs are pivotal in regulating inflammation levels through multiple mechanisms, influencing CNS inflammation during PD onset and progression.

In addition to SCFAs, numerous microbial-derived metabolites have recently been implicated in the pathogenesis of PD. Aromatic amine metabolites such as isoamylamine, predominantly produced by bacteria belonging to the family Ruminococcaceae, can cross the blood-brain barrier and activate the S100A8–p53 signaling pathway, thereby inducing microglial apoptosis and affecting immune homeostasis in the CNS.^85^ Metabolites derived from tryptophan metabolism are also critically involved in maintaining intestinal barrier integrity,^86^^,^^87^ Among them, indole derivatives, such as indole-3-propionic acid, have been shown to exert neuroprotective effects by activating the aryl hydrocarbon receptor (AHR) pathway, consequently inhibiting aberrant microglial activation.^88^^,^^89^ Bile acids, another class of important microbiota-host co-metabolites, have recently attracted considerable attention in the context of neurodegenerative diseases. Certain secondary bile acids, including tauroursodeoxycholic acid (TUDCA) and ursodeoxycholic acid (UDCA), exhibit antioxidative, anti-apoptotic, and anti-inflammatory properties and may beneficially modulate mitochondrial function and neuroinflammation in PD.^90^^,^^91^ Additionally, trimethylamine N-oxide (TMAO), produced from choline metabolism by gut microbiota, has been demonstrated to enhance microglial activation and upregulate pro-inflammatory cytokine expression, although its potential pathogenic role in PD remains to be further clarified.^92^^,^^93^ In summary, these gut microbial metabolites modulate neuroimmune responses and cellular homeostasis via multiple signaling pathways, providing a novel metabolomic perspective for understanding PD pathogenesis.

Beyond bacteria, recent population-based and multi-omics investigations suggest that the intestinal mycobiome and virome are also associated with PD, although the evidence remains inconclusive. A cross-sectional study using fungal-specific internal transcribed spacer 2 (ITS2) amplicon sequencing detected no overall differences in the fecal mycobiota between PD and control groups^94^; by contrast, another study that profiled fungal rRNA alongside the fecal metabolome reported reduced SCFAs in PD, accompanied by increases in *Hanseniaspora*, *Kazachstania*, Tremellaceae, and *Penicillium* with a concomitant decrease in *Saccharomyces*, suggesting that fungi may influence PD through metabolite-mediated pathways.^95^ Given the low abundance and highly unstable colonization of intestinal fungal communities,^96^ and the observation that the most frequently detected taxa are common food- or environment-associated fungi (with only *Saccharomyces* and *Candida* capable of surviving at body temperature),^94^ these findings may reflect short-term gut health rather than a PD-specific mycobiome dysbiosis.

With respect to the gut virome, current evidence indicates predominance of bacteriophages: early work in medication-naive PD cohorts found the enrichment of lytic lactococcal phages (c2/936-like) in stool together with the marked depletion of the host genus *Lactococcus*, implying that phage-driven bacterial imbalance could compromise barrier integrity and perturb local metabolic pathways.^97^ Subsequent, deeper metagenomic analyses have identified detectable differences in the intestinal phageome and plasmidome between PD and controls, although these observations remain exploratory and await multicenter validation.^61^ In gut-initiated PD models, the injection of α-synuclein or preformed fibrils (PFFs) into the enteric nervous system induced long-term remodeling of the fecal virome, an effect amplified by LPS, suggesting links between virome alterations and host pathological protein changes.^98^ Studies of the gut virome are constrained by pronounced inter-individual variability, incomplete reference databases, and biases introduced by virus-like particle (VLP) enrichment and multiple displacement amplification (MDA); moreover, low-biomass samples are susceptible to contamination and artifacts from host-depletion steps, making null or contradictory findings not uncommon.^99^ Future work will require more advanced methodologies and standardized workflows to elucidate the roles of intestinal fungal and viral communities in the pathogenesis of PD.

In summary, gut microbiota dysbiosis impairs intestinal barrier function, activates the host immune system, and triggers systemic inflammation, playing a crucial role in CNS inflammation associated with PD. Recently, the widespread application of 16S rRNA and 18S rRNA amplicon sequencing techniques has greatly advanced research on gut microbial structural alterations in patients with PD. Compared to metagenomic sequencing, these amplicon sequencing approaches offer advantages such as lower cost and higher efficiency. However, they still lack in-depth functional characterization and fail to accurately capture complex microbial interactions, resulting in discrepancies regarding specific microbial changes across studies. Thus, future research must focus on more detailed, comprehensive functional analyses to further elucidate the precise roles of gut microbiota dysbiosis in PD pathogenesis.

### Propagation and regulation mechanisms of pathological α-syn aggregation in the gut-brain axis

α-Synuclein, the hallmark pathological protein of Parkinson’s disease (PD), has long been a focus of investigation. Intestinal dysbiosis and its products—such as bacterial amyloids (e.g., curli) and LPS—promote α-syn aggregation within the gut and exacerbate motor deficits and neuroinflammation,^29^ indicating that the intestinal microenvironment can accelerate PD progression by fostering gut α-syn pathology. Moreover, models in which α-syn ascends *trans*-synaptically along the vagus nerve to the medulla and midbrain have been validated by numerous studies, and interventions targeting the vagal pathway, such as vagotomy, impede this ascending propagation,^100^ further supporting this mechanism. In summary, diverse gut-derived factors can mediate and amplify α-syn pathology, thereby influencing the course of disease progression.

#### Pathophysiological roles of α-synuclein in Parkinson’s disease

α-syn, a member of the synuclein family, is composed of 140 amino acids and is highly conserved across species. It is extensively expressed throughout the CNS and peripheral nervous system (PNS), especially enriched at presynaptic terminals of neurons.^101^^,^^102^ Encoded by the SNCA gene, α-syn is an intrinsically disordered protein, capable of adopting various conformations, including the monomeric unstructured form in the cytoplasm, α-helical structure upon membrane-binding, and pathological oligomeric and fibrillar forms rich in β-sheet structures. These fibrillar forms pathologically deposit into Lewy bodies (LBs) and Lewy neurites (LNs), which constitute the hallmark pathological features of PD.^101^^,^^103^

Under physiological conditions, α-syn participates in regulating the synaptic vesicle cycle, including vesicle clustering, docking, SNARE complex assembly, and maintenance of vesicle pools.^104^ Studies on α-syn knockout animal models have revealed reductions in the number of docked vesicles and impaired replenishment from reserve vesicle pools to docked pools, leading to impaired synaptic transmission during prolonged stimulation.^105^ Furthermore, α-syn interacts simultaneously with vesicle-associated membrane protein 2 (VAMP2/synaptobrevin-2) and synaptic vesicle phospholipids, facilitating SNARE complex assembly, which is critical for vesicle fusion and neurotransmitter release.^106^^,^^107^

LB pathology was first discovered in the ENS of patients with PD by Qualman et al. in 1984, specifically within the colonic Auerbach plexus and esophageal ganglion cells, with accompanying neuronal degeneration in the latter.^108^ Subsequent studies by Wakabayashi et al. further confirmed the widespread presence of LB pathology throughout the ENS, extending from the esophagus to the rectum within both the Auerbach and Meissner plexuses.^109^^,^^110^^,^^111^ It was not until 1997 that α-syn was clearly identified as the principal constituent of LBs.^112^ The core pathological features of PD include the progressive loss of dopaminergic neurons in the SNpc, accompanied by abnormal α-syn aggregation within LBs and LNs.^113^ Although α-syn aggregation is widely acknowledged as central to PD pathogenesis, the specific initiating factors and mechanisms of propagation remain incompletely understood. Rare missense mutations in the SNCA gene (e.g., A30P and A53T) have been associated with familial PD, significantly altering the aggregation properties of α-syn.^114^^,^^115^ Additionally, post-translational modifications of α-syn, particularly phosphorylation at Ser129, critically influence its aggregation and clearance, playing pivotal roles in the pathological progression of PD.^116^^,^^117^ Currently, accumulating evidence supports that pathological α-syn aggregates contribute to PD by interfering with mitochondrial functions, disrupting calcium homeostasis, impairing proteostasis, and eliciting abnormal immune responses,^118^^,^^119^ Meanwhile, aggregated α-syn disrupts synaptic vesicle cycling, ultimately leading to neuronal dysfunction and degeneration, thereby driving PD progression and clinical symptom emergence.^120^^,^^121^

#### Pathways and mechanisms of α-synuclein propagation via the gut-brain axis

Numerous studies have confirmed pathological α-syn aggregation in the ENS of patients with PD, which may precede similar pathology in the CNS.^7^ However, whether α-syn aggregation in the gut definitively propagates to the brain remains uncertain. Incidental Lewy body disease (ILBD), considered a precursor stage of PD, shows widespread LB pathology throughout the digestive tract, including the esophagus, stomach, small intestine, and colon, as indicated by autopsy studies.^122^^,^^123^ Stokholm et al. reported phosphorylated α-syn pathology in gut neurons in 56% of individuals during the prodromal stage of PD, occurring as early as two decades before clinical diagnosis.^124^ Nevertheless, this evidence alone does not confirm that pathology necessarily originates in the ENS, nor does it clarify the precise mechanisms by which pathological α-syn propagates to the CNS.

The Braak hypothesis proposes that PD pathology may begin in either the GI tract or olfactory system and subsequently propagate to the CNS via the VN.^6^^,^^7^ Animal studies support this view: Injection of α-syn PFFs into the gut induces α-syn aggregation within the gut and vagal nerve, with subsequent spread to the CNS; vagotomy delays pathological progression and onset of motor dysfunction.^125^^,^^126^ Uemura et al. demonstrated that the injection of PFFs into the gastric wall of transgenic mice led to the detection of phosphorylated α-syn in the dorsal motor nucleus of the vagus (DMN), which only occurred on the intact (non-vagotomized) side, providing strong evidence that vagus nerve transection blocks pathological transmission.^127^^,^^128^ Additionally, oral administration of PFFs also induced LB pathology in the CNS.^129^^,^^130^

α-Syn propagation demonstrates prion-like characteristics, capable of self-replication by “seeding,” whereby misfolded α-syn induces the aggregation of native α-syn, amplifying pathological burden.^131^^,^^132^ Moreover, α-syn aggregates can spread along neuronal axons over long distances and propagate transcellularly.^133^ LB-like pathology detected in fetal midbrain neurons transplanted into patients with PD further supports the notion of intercellular α-syn propagation in humans.^134^^,^^135^ Epidemiological studies suggesting reduced PD risk following vagotomy further support this propagation model, although these findings remain controversial.^136^^,^^137^^,^^138^

Some research has proposed that α-syn propagation may also occur independently of the VN. For example, Arotcarena et al. demonstrated that gut-injected PFFs induced nigrostriatal pathology in a non-human primate model without detectable vagal nerve involvement, suggesting a possible systemic circulatory transmission.^139^ Other studies have proposed non-neural propagation pathways for α-syn, including transmission through the celiac ganglion or via erythrocyte-derived extracellular vesicles.^126^^,^^140^ Therefore, pathways of α-syn propagation remain debated, necessitating further clarification through diverse animal models.

#### Regulatory roles of gut microbiota, inflammation, and enteroendocrine cells on α-synuclein aggregation and propagation via the gut-brain axis

Pathological α-syn aggregates induce neuroinflammation by activating microglia and promoting M1 polarization, leading to the production of inflammatory cytokines and reactive oxygen species (ROS), ultimately causing neuronal damage.^141^ The gut, as a potential initial site for α-syn pathology, has attracted considerable attention. Studies have indicated that certain gut microbes can trigger or enhance α-syn aggregation.^142^ For example, bacterial amyloids derived from biofilm-associated proteins (BAPs) produced by gut microbes can induce α-syn aggregation in Caenorhabditis elegans models and dopamine neurons.^143^ Similarly, purified curli protein, expressed by Escherichia coli, accelerates α-syn aggregation and forms a bidirectional cross-seeding effect with α-syn, exacerbating pathological progression.^29^^,^^144^ Additionally, Desulfovibrio species have been reported to promote α-syn aggregation and induce PD-like pathology in C. elegans models.^145^ Non-amyloid proteins derived from bacteria may also facilitate α-syn misfolding and aggregation by inducing oxidative stress or directly interacting with host proteins.^146^ Furthermore, intestinal fatty acid-binding protein (FABP2) co-localizes with α-syn, and its plasma levels correlate with disease progression in PD, suggesting its involvement in α-syn uptake and aggregation as a potential disease biomarker.^147^ Recent findings demonstrate that bacterial endotoxins activate the C/EBPβ-AEP pathway, promoting α-syn cleavage at the N103 site by asparagine endopeptidase (AEP), thereby accelerating α-syn aggregation and neurotoxicity.^148^^,^^149^

Intestinal inflammation significantly influences α-syn aggregation and propagation.^150^ On one hand, inflammatory mediators promote abnormal expression and misfolding of α-syn; on the other hand, inflammation increases gut barrier permeability, facilitating the peripheral dissemination of α-syn.^151^^,^^152^ Thus, suppressing intestinal inflammation and protecting gut barrier integrity represent potential strategies for delaying PD progression. SCFAs, particularly butyrate, are essential for maintaining gut barrier integrity,^153^ Reduced SCFA availability increases susceptibility for α-syn to propagate to the brain via the gut-brain axis.^142^

EECs, comprising approximately 1% of intestinal epithelial cells, include diverse subtypes such as gastrin-secreting G cells, cholecystokinin-producing I cells, and L cells secreting glucagon-like peptide-1 (GLP-1) and peptide YY (PYY).^154^^,^^155^ EECs are positioned with apical surfaces oriented toward the gut lumen and basal surfaces closely connected to neural plexuses, sensing luminal changes and influencing brain function through hormonal and neurotransmitter secretion.^156^ Studies have revealed that EECs also express α-syn.^157^ Intestinal inflammation disrupts EEC functions and promotes pathological α-syn aggregation and neuroinflammation.^158^ Recent research further shows that α-syn expressed in EECs can transfer to enteric neurons and subsequently propagate to the CNS via the VN.^159^ Therefore, investigation of EECs provides novel insights into α-syn propagation from the gut, offering new evidence and therapeutic avenues regarding the gut-brain axis in PD pathogenesis.

### Mitochondrial dysfunction induced by gut-related factors in Parkinson’s disease

An increasing body of research identifies mitochondrial dysfunction as a pivotal event in the pathogenesis of PD. Its core phenotypes—elevated oxidative stress, dysregulated mitophagy, and bioenergetic insufficiency—collectively heighten the vulnerability of dopaminergic neurons.^160^ Gut-derived factors drive mitochondrial stress along the gut-brain axis: dysbiosis and barrier disruption permit endotoxin (lipopolysaccharide, LPS) to enter the circulation or act directly on the enteric nervous system, triggering inflammatory oxidative responses and impairing mitochondrial function.^161^ Concurrently, abnormalities in microbial metabolites—most notably SCFAs and bile acid profiles—disrupt mitochondrial oxidative metabolism and autophagy homeostasis, thereby promoting PD progression.^76^^,^^162^ Accordingly, the microbiota-metabolite-mitochondrion axis constitutes a promising target for future therapeutic interventions.

#### Role of mitochondrial dysfunction in Parkinson’s disease pathology

Mitochondria are the primary source of cellular energy, generating adenosine triphosphate (ATP) through oxidative phosphorylation,^163^and play critical roles in regulating cell apoptosis and immune responses.^164^ Mitochondrial dysfunction can result not only in impaired ATP production but also in the excessive generation of ROS, which consequently triggers oxidative stress and neuronal damage.^165^ Furthermore, mitochondrial impairment may lead to the release of inflammatory mediators, thereby activating immune cells and exacerbating neuroinflammation.^166^ Thus, perturbations in energy metabolism and increased oxidative stress constitute critical mechanisms underlying neurodegeneration.^167^

In PD, mitochondrial complex I activity within substantia nigra neurons is markedly reduced by approximately 35%, limiting ATP production and elevating ROS levels, ultimately promoting dopaminergic neuron damage and apoptosis.^168^^,^^169^ Defects in mitochondrial quality control also contribute significantly to PD pathology, particularly via the impairment of the PINK1-Parkin signaling pathway. Dysfunction in this pathway compromises mitophagy, resulting in the accumulation of damaged mitochondria and subsequent aggravation of neuronal injury.^170^ In addition, the aggregation of α-syn has been demonstrated to inhibit complex I activity, thereby intensifying mitochondrial dysfunction and establishing a vicious cycle that accelerates PD progression.^171^

#### Impact of gut microbiota and their metabolites on mitochondrial function and autophagy

In recent years, growing attention has been focused on the influence of the gut microbiota in the development and progression of PD. The gut microbiota can adversely affect mitochondrial function via several distinct mechanisms. On one hand, microbial metabolites such as SCFAs, bile acids, and polyphenolic compounds can modulate host mitochondrial functions.^172^ For instance, butyrate can enhance mitochondrial respiratory chain activity and mitigate oxidative stress,^173^ whereas bile acids influence mitochondrial membrane integrity and neuronal survival.^172^ On the other hand, gut dysbiosis results in increased production of harmful substances such as LPS, which upon entry into the CNS, induce mitochondrial dysfunction and oxidative damage.^174^

Chronic gut inflammation associated with microbiota dysbiosis releases cytokines and ROS, causing direct damage to mitochondrial DNA and proteins, thereby impairing mitochondrial function.^175^ Additionally, intestinal inflammatory signals can be transmitted to the brain via vagal nerve and systemic circulation, further exacerbating mitochondrial impairment and oxidative stress in neurons.^176^ Furthermore, gut microbiota may enhance host susceptibility to environmental toxins; for example, pesticides such as rotenone and paraquat can transit from gut lumen to the brain, directly inhibiting mitochondrial complex I activity, inducing mitochondrial dysfunction and accumulating ROS.^173^ The gut microbiota are also capable of synthesizing or metabolizing neurotoxic substances such as precursors of the neurotoxin MPTP^175^ and certain metabolites derived from Streptomyces species, all of which may increase ROS, disrupt mitochondrial homeostasis, aggravate α-syn aggregation, and promote neuronal injury.^177^ Additionally, microbial-derived hydrogen sulfide (H_2_S) can stimulate mitochondrial cytochrome *c* release and ROS production, further promoting the pathological accumulation of α-syn.^178^

Mitophagy, the selective degradation of damaged mitochondria, represents a crucial mechanism for maintaining mitochondrial quality. Gut microbiota dysbiosis has been implicated in the disruption of the PINK1-Parkin pathway, compromising mitophagy, accumulating damaged mitochondria, and increasing ROS levels, ultimately accelerating neurodegeneration.^172^^,^^176^ Mutations in the PINK1 and Parkin genes have been reported in both familial and sporadic PD cases, underscoring the significance of this pathway in disease pathogenesis.^172^

In conclusion, mitochondrial dysfunction represents a central pathogenic mechanism in PD. Gut microbiota dysbiosis contributes to the onset and progression of PD by disrupting mitochondrial energy metabolism, exacerbating oxidative stress, promoting inflammatory responses, and impairing mitophagy.

### Potential therapeutic strategies targeting gut microbiota in Parkinson’s disease: current progress and future perspectives

**Figure 3:**
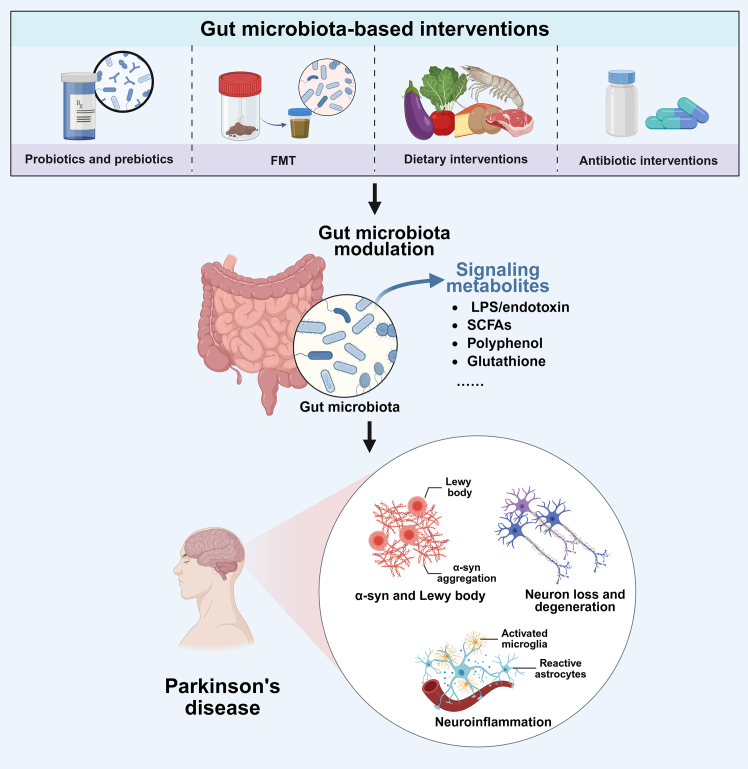
Gut microbiota-based interventions targeting Parkinson’s disease Probiotics and prebiotics, fecal microbiota transplantation, dietary modulation, and antibiotic interventions reshape gut microbiota and metabolites to modulate α-syn pathology, neuroinflammation, and neuronal degeneration.

Given the intimate relationship between gut microbiota and PD, therapeutic strategies targeting gut microbiota have demonstrated considerable potential. With advancements in the understanding of the gut-brain axis, numerous studies have explored methods to ameliorate PD symptoms via the modulation of intestinal functions. Among these approaches, probiotics, prebiotics, and FMT have attracted considerable attention, with Tables 2, 3, and 4 summarizing key findings from related animal model studies. Additionally, dietary interventions and antibiotic treatments have also shown promise by reshaping gut microbiota composition, thereby potentially alleviating PD symptoms. Figure 3 provides an overview of these gut microbiota-based interventions.

**Table 2:** Summary of studies investigating probiotic interventions for PD

Probiotic Strains	Intervention Duration	Key Findings	Reference
Lactobacillus plantarum (CCFM405)^179^	Daily, 8 weeks	Increased colonic expression of tight junction proteins (Occludin, Zonula Occludens-1); reduced pro-inflammatory cytokines in colon and brain; inhibited activation of microglia and astrocytes in substantia nigra; alleviated dopaminergic neuron damage and improved motor deficits in rotenone-induced PD mice.	Chu et al.^179^ (2023)
Lactobacillus plantarum (PS128)^180^	Daily, 4 weeks	Suppressed activation of microglia and astrocytes in striatum; decreased TNF-α levels; reduced striatal and midbrain dopaminergic neuronal loss; ameliorated motor dysfunction in MPTP-induced PD mice.	Liao et al.^180^ (2020)
Bifidobacterium breve (CCFM1067)^181^	Daily, 33 days	Increased colonic tight junction protein expression (Occludin, Zonula Occludens-1, Claudin-1); elevated fecal SCFA levels (acetic and butyric acids); suppressed microglial and astrocytic activation in the striatum; alleviated motor dysfunction in MPTP-induced PD mice.	Li et al.^181^ (2022)
Lactococcus lactis (MG1363-pMG36e-GLP-1)^182^	Daily, 7 days	Enhanced expression of tight junction proteins (Occludin, Zonula Occludens-1) in colon and substantia nigra; activated Keap1-Nrf2-GPX4 signaling pathway to inhibit ferroptosis in substantia nigra; reduced α-syn aggregation; improved motor dysfunction in MPTP-induced PD mice.	Yue et al.^182^ (2022)
Probiotic cocktail (Lactobacillus rhamnosus GG, Lactobacillus acidophilus, Lactobacillus plantarum)^183^	Daily, 30 days	Inhibited activation of microglia and astrocytes in substantia nigra; reduced dopaminergic neuron loss in substantia nigra and striatum; alleviated motor deficits in MPTP and rotenone-induced PD mouse models.	Srivastav et al.^183^ (2019)

**Table 3:** Summary of studies investigating prebiotic interventions for PD

Prebiotic Intervention	Intervention Duration	Key Findings	Reference
Galacto-oligosaccharide (GOS)^184^	Daily, 4 weeks	Increased fecal SCFAs; elevated expression of tight junction proteins (Occludin, Zonula Occludens-1) in colon and substantia nigra; reduced serum pro-inflammatory cytokines (TNF-α and IL-6); attenuated dopaminergic neuronal loss in striatum and cortex; alleviated motor deficits in MPTP-induced PD mice.	Dong et al.^184^ (2020)
High-fiber prebiotic dietary intervention^185^	Daily, 7 weeks	Enhanced fecal SCFAs levels (acetate, propionate, butyrate, isobutyrate); suppressed activation of microglia in substantia nigra and striatum; upregulated expression of TREM2 (a neuroprotective microglial phenotype marker); reduced α-syn aggregation in substantia nigra; improved motor function in PD mice.	Abdel-Haq et al.^185^ (2023)
Combination of Fructooligosaccharide (FOS) and Lactobacillus rhamnosus GG (Synbiotic)^186^	Daily, 5 weeks	Compared to prebiotic or probiotic alone, synbiotic increased expression of tight junction proteins (Occludin, Zonula Occludens-1) in the striatum; attenuated dopaminergic neuronal loss in midbrain and striatum; improved motor deficits in MPTP-induced PD mice.	Liu et al.^24^^,^^186^ (2022)

**Table 4:** Summary of studies investigating FMT interventions for PD

Donor Source	Intervention Duration	Key Findings	Reference
Healthy control mice^187^	Daily, 7 days	Suppressed the TLR4/TBK1/NF-κB/TNF-α signaling pathway in colon and striatum; reduced activation of microglia and astrocytes in substantia nigra and striatum; alleviated dopaminergic neuronal loss in substantia nigra; improved motor deficits in MPTP-induced PD mice.	Sun et al.^187^ (2018)
Healthy human donors^188^	Daily, 10 days	Enhanced expression of tight junction protein Zonula Occludens-1 in colon; reduced expression of pro-inflammatory cytokine IL-1β in colon and striatum; activated the AMPK/SOD2 signaling pathway in colon and substantia nigra, thus reducing the apoptosis of peripheral glial cells; alleviated gut-brain dysfunction; improved motor deficits and attenuated dopaminergic neuronal loss in striatum in MPTP-induced PD mice.	Xie et al.^188^ (2023)
Healthy control mice^189^	Daily, 7 days	Suppressed the TLR4/PI3K/AKT/NF-κB signaling pathway in the striatum; inhibited microglial activation in substantia nigra; reduced dopaminergic neuronal loss; decreased α-syn aggregation in substantia nigra; improved motor deficits in MPTP-induced PD mice.	Zhong et al.^189^ (2021)
Healthy control mice^50^	Daily, 2 weeks	Increased expression of tight junction proteins (Occludin, Zonula Occludens-1, Claudin-1) in colon and substantia nigra; alleviated motor dysfunction induced by rotenone; reduced serum levels of lipids and pro-inflammatory cytokines (IL-6, IL-1β, TNF-α); suppressed TLR4/MyD88/NF-κB signaling pathway in colon and substantia nigra; alleviated dopaminergic neuronal loss; reduced α-syn aggregation; improved motor function in rotenone-induced PD mice.	Zhao et al.^50^ (2021)

#### Advances in probiotics and prebiotics in alleviating Parkinson’s disease symptoms through the modulation of gut microbiota

Probiotics and prebiotics have recently emerged as critical tools for modulating gut microbiota, demonstrating substantial therapeutic potential in neurodegenerative diseases. Probiotics refer to live microorganisms that confer health benefits to the host when administered in adequate amounts.^190^ In addition to traditional Lactobacillus and Bifidobacterium strains, emerging probiotics such as *Akkermansia muciniphila*, *Faecalibacterium prausnitzii*, and *Bacteroides fragilis* have increasingly gained attention.^191^^,^^192^^,^^193^ These probiotic strains are capable of producing abundant SCFAs, which enhance intestinal barrier integrity, regulate immune responses, and attenuate neuroinflammation, consequently slowing PD progression. Randomized, double-blind, controlled trials (RCTs) increasingly suggest that probiotics may alleviate constipation in PD. In a 4-week RCT (*n* = 72), Tan et al. demonstrated that a multi-strain probiotic capsule significantly increased the number of weekly spontaneous bowel movements (SBMs) in patients with PD, and improved stool consistency and constipation-related quality of life, although fecal calprotectin showed no significant change.^194^ Similarly, in an 8-week randomized controlled study (*n* = 48), Ibrahim et al. found that multi-strain probiotics increased bowel-movement frequency, shortened whole-gut transit time, and were well tolerated.^195^ Meta-analyses pooling multiple RCTs generally indicate that probiotics increase weekly SBMs in PD; however, heterogeneity persists for outcomes such as stool consistency, abdominal bloating, and overall quality of life, yielding a low overall certainty of evidence and underscoring the need for longer-duration trials with standardized regimens.^196^ Collectively, probiotic interventions in PD appear to consistently improve constipation-related endpoints (e.g., SBMs), whereas effects on motor and broader non-motor symptoms are comparatively modest and heterogeneous.

Prebiotics, non-digestible dietary components that selectively promote beneficial gut microbiota growth or activity—such as inulin, fructo-oligosaccharides (FOS), and galacto-oligosaccharides (GOS)—have also shown therapeutic promise.^197^^,^^198^ Prebiotics facilitate the proliferation of specific microbial populations, indirectly elevating SCFA production, suppressing intestinal inflammation, improving gut barrier function, and consequently mitigating neuroinflammation and PD pathology.^197^ Animal studies have demonstrated that both probiotics and prebiotics effectively reduce systemic endotoxin (e.g., LPS) translocation, diminishing peripheral and CNS inflammatory responses and delaying neurodegeneration.^190^^,^^191^^,^^192^ For instance, butyrate produced by *Faecalibacterium prausnitzii* exhibits potent anti-inflammatory effects, protects gut and blood-brain barriers, and alleviates PD-related neuroinflammation.^191^ Hall et al. conducted an open-label, nonrandomized, short-term (10-day) intervention in patients with PD using a mixed prebiotic fiber formulation (resistant starch, resistant maltodextrin, inulin, and rice bran), enrolling newly diagnosed, drug-naive participants (*n* = 10) and treated patients (*n* = 10); the primary endpoint was tolerability and the secondary endpoint was safety. The intervention was well tolerated and safe, and was associated with increases in fecal and plasma SCFAs, reductions in fecal calprotectin, and a modest decrease in serum neurofilament light chain (NfL).^199^ Growing clinical interest has also focused on synbiotic preparations (probiotics combined with prebiotics) in PD. In an open-label, single-arm Italian study, 12 weeks of synbiotic therapy (Enterolactis Duo; containing the probiotic strain *Lacticaseibacillus paracasei* DG and the prebiotic fiber inulin) significantly improved stool consistency, increased fecal *Faecalibacterium prausnitzii*, and raised the butyrate/acetate ratio; scores on the Movement Disorder Society-Unified Parkinson’s Disease Rating Scale (MDS-UPDRS) Part I and the Scales for Outcomes in Parkinson’s disease-Autonomic (SCOPA-AUT) also decreased. However, the absence of a control group and limited stool sampling necessitate confirmation in larger RCTs.^200^ A recent RCT evaluated inulin plus *Lactobacillus acidophilus* as adjunctive therapy to levodopa for 3 months; relative to controls, the synbiotic group exhibited greater improvements across MDS-UPDRS subscales, accompanied by decreases in serum TNF-α and malondialdehyde (MDA) and an increase in brain-derived neurotrophic factor (BDNF), suggesting potential anti-inflammatory, antioxidant, and neurotrophic effects. Nonetheless, the trial was unblinded and of short duration, underscoring the need for double-blind, multicenter studies to establish robustness and reproducibility.^201^

In conclusion, despite promising results from animal models, clinical findings on probiotics and prebiotics remain complex and inconsistent. Although generally considered safe, their therapeutic efficacy exhibits significant inter-individual variability closely linked to the patients’ baseline microbiota profiles.^202^^,^^203^ Accordingly, future research should prioritize personalized therapeutic strategies that tailor probiotic, prebiotic, or synbiotic interventions to the clinical profiles of patients with Parkinson’s disease. These approaches must be underpinned by well-powered, multicenter, rigorously blinded, high-quality randomized controlled trials, supported by reproducible clinical and mechanistic endpoints.

#### Current status and clinical challenges of fecal microbiota transplantation in Parkinson’s disease treatment

FMT, involving the transfer of fecal microbiota from healthy donors to restore microbial balance, has emerged as an innovative strategy for ameliorating gut dysbiosis.^204^^,^^205^ Animal model studies have shown that FMT can restore gut and blood-brain barrier functions, reduce systemic and neuroinflammation, and alleviate PD pathology.^206^ Currently, clinical research on FMT for PD is still in its preliminary stages; although certain studies indicate improvements in gut microbiota composition and constipation symptoms, overall therapeutic efficacy and long-term stability remain uncertain.^205^ Multiple factors, including donor and recipient microbial characteristics, transplantation methodology, gender, age, and general health status, significantly influence the clinical outcomes of FMT.^207^ A double-blind RCT from Finland indicates that FMT is safe for patients with PD but does not ameliorate PD symptoms.^208^ In contrast, another randomized, double-blind, placebo-controlled clinical study showed that FMT significantly reduced constipation in PD, potentially attributable to increases in two beneficial bacterial families—Lachnospiraceae and Lactobacillaceae.^209^ Notably, multiple clinical studies in functional bowel disorders suggest that female patients with PD may be more likely to respond to FMT; comparable sex differences have been observed in other FMT-related trials. In a double-blind RCT of patients with bloating-predominant irritable bowel syndrome (IBS), subgroup and long-term follow-up analyses indicated higher overall response and maintenance rates in women, and baseline microbiome characteristics correlated with treatment efficacy, implying that sex differences may be mediated indirectly through microbial features.^210^ Another IBS study further found that increasing the transplant dose or employing repeated FMT enabled men to achieve overall response rates comparable to those of women, suggesting that dosing intensity and administration frequency can partially offset sex-related baseline differences.^211^ In addition, systematic reviews have identified sex as a putative effect modifier that warrants prospective validation.^212^ Mechanistically, current evidence supports the “microgenderome” concept, whereby sex hormones and immune differences, bile-acid and short-chain fatty-acid metabolism, and intestinal barrier and motility exhibit sex-dimorphic features that influence donor-recipient microbial engraftment and strain-level competition.^213^ Future studies in PD should therefore account for sex as a modifier; for male participants, repeated administration or higher-dose strategies may be prioritized, and a broader panel of biomarkers—such as gastrointestinal barrier integrity and inflammatory indices—should be incorporated to assess efficacy and reduce sex-related heterogeneity in treatment response.

Moreover, to enhance therapeutic outcomes, current studies have investigated optimized donor selection criteria, pretreatment regimens, and innovative transplantation technologies such as microencapsulation and nanoparticle encapsulation to improve microbiota viability and intestinal targeting.^214^^,^^215^ Personalized precision interventions targeting microbiota also represent critical future research directions.^216^ Overall, compared with probiotics and prebiotics, FMT exhibits less consistent efficacy; clinical outcomes are influenced by heterogeneity in the route of administration, dosing frequency, donor selection and manufacturing processes, recipient baseline microbiota and medication exposure, and bowel-preparation protocols, and placebo effects are not negligible. While overall safety in trials involving patients with Parkinson’s disease appears acceptable, substantial heterogeneity persists across studies.

In conclusion, several challenges remain in the clinical implementation of probiotics, prebiotics, and FMT. Existing clinical trials often lack detailed analyses of microbiota shifts pre- and post-treatment, limiting comprehensive assessments of efficacy.^202^^,^^203^ Additionally, probiotic and prebiotic viability can be compromised by gastric acidity and digestive enzymes.^214^ Furthermore, FMT faces issues related to low transplant success rates, donor selection difficulties, and a lack of standardized treatment protocols.^205^^,^^212^ Advances in microbiome research and precision medicine are anticipated to foster breakthroughs in PD therapies, integrating cutting-edge approaches such as gene editing and nanotechnologies to facilitate personalized microbiome modulation.

#### Dietary interventions (Mediterranean and ketogenic diets) as preventive and therapeutic strategies in Parkinson’s disease

Dietary interventions have recently garnered significant attention for their potential roles in preventing and managing PD.^217^^,^^218^^,^^219^^,^^220^ Diet can influence PD onset and progression by modulating gut microbiota composition and related metabolites via the gut-brain axis, with the Mediterranean diet (MD) and ketogenic diet (KD) showing particular promise.

The Mediterranean diet, characterized by high dietary fiber and unsaturated fat intake, emphasizes fruits, vegetables, cereals, fish, and moderate wine consumption, while limiting red meat and saturated fats.^220^^,^^221^ Epidemiological studies indicate an inverse correlation between adherence to MD and PD incidence.^222^ Dietary fibers within the MD facilitate the growth of beneficial bacteria producing SCFAs, thus suppressing intestinal inflammation and strengthening barrier integrity.^223^ Additionally, MD is rich in polyphenolic antioxidants, which may restore oxidative homeostasis, mitigate neuroinflammation, and promote α-syn clearance, further reducing PD risk.^224^^,^^225^

The ketogenic diet, characterized by high fat, moderate protein, and low carbohydrate content, induces ketogenesis as an alternative energy source to glucose.^226^ Clinical trials have demonstrated that a KD administered for 4–8 weeks significantly improves motor and non-motor symptoms in patients with PD.^227^ Animal models of MPTP-induced PD also support ketones’ neuroprotective efficacy in safeguarding dopaminergic neurons and ameliorating motor deficits.^228^^,^^229^ Proposed mechanisms include increasing glutathione levels, enhancing antioxidant capacity, optimizing energy metabolism, and exerting anti-inflammatory effects.^230^^,^^231^^,^^232^

Overall, dietary interventions represent promising therapeutic approaches for PD, yet further research is required to clarify active dietary components and underlying mechanisms. Limited clinical evidence and challenges regarding long-term patient adherence, particularly with KD-associated discomfort, remain significant obstacles to their widespread adoption.

#### Antibiotic interventions for Parkinson’s disease treatment: Research progress and clinical controversies

Recently, antibiotics have attracted attention in PD research due to their antimicrobial, anti-inflammatory, and neuroprotective effects.^233^^,^^234^ In an MPTP-induced PD mouse model, vancomycin administration increased the abundance of beneficial bacteria such as *Blautia* and *Akkermansia muciniphila*, alleviating neuroinflammation via the inhibition of the TLR-4/NF-κB pathway.^13^^,^^235^ Despite clinical evidence suggesting that increased *Akkermansia muciniphila* in patients with PD may impair intestinal barrier integrity,^13^^,^^174^^,^^236^ such findings highlight the importance of maintaining microbial homeostasis.

Ceftriaxone, a β-lactam antibiotic with established safety profiles,^237^^,^^238^ similarly increases *Akkermansia muciniphila* abundance and mitigates symptoms in MPTP-induced PD mice.^239^ Subsequent studies confirmed that ceftriaxone reduces pro-inflammatory cytokines, including TLR-4, MyD88, NF-κB, IL-1β, and TNF-α expression in colon and brain tissues.^240^^,^^241^ Furthermore, *in vitro* studies identified a high affinity between ceftriaxone and α-syn, thereby preventing its aggregation.^238^^,^^242^ The broad-spectrum antibiotic rifampicin also demonstrates therapeutic potential in various PD animal models, enhancing tyrosine hydroxylase (TH) expression, reducing oxidative stress, restoring dopaminergic signaling, and suppressing neuroinflammation.^243^^,^^244^^,^^245^

Other antibiotics, such as doxycycline, minocycline, and rifaximin, have exhibited promising effects in PD models.^246^^,^^247^^,^^248^ However, discrepancies remain between clinical and preclinical antibiotic data, alongside concerns regarding antibiotic-induced gut dysbiosis, antibiotic resistance, and potential adverse effects.^144^^,^^249^ Epidemiological evidence indicates long-term antibiotic use may even increase PD risk.^250^ Thus, future research should optimize experimental design and models to comprehensively and cautiously assess the efficacy and safety of antibiotics as PD treatments, emphasizing microbiota functional profiling and personalized therapeutic strategies.

## Discussion

PD, a prevalent neurodegenerative disorder, has recently experienced a paradigm shift from traditional CNS-centric perspectives toward the gut-brain axis. Although PD has historically been classified as a brain disorder, emerging evidence highlights the significance of complex bidirectional interactions between the gut and the brain, wherein the gut microbiota appears to play a pivotal role. Dysbiosis in patients with PD is frequently characterized by reductions in beneficial bacterial genera such as *Prevotella*, coupled with increases in pathogenic genera such as *Clostridium*, along with decreased SCFAs levels, exacerbating inflammatory responses both locally within the gut and systemically within the CNS. Current research has predominantly emphasized compositional profiling of the intestinal microbiota; future investigations should more broadly interrogate microbial functions and their complex interactions to delineate the precise influence of the gut microbiome on the pathophysiology of Parkinson’s disease.

Pathological aggregation of α-syn constitutes a hallmark feature of PD. Accumulating evidence suggests that pathological changes in α-syn may originate or become amplified within the gut. Consistent with this, Braak’s hypothesis proposes that α-syn pathology initiates in the gut and subsequently propagates to the CNS via the VN. Nevertheless, the precise neural and immune pathways facilitating α-syn transmission from the gut to the brain remain incompletely understood. Future investigations are warranted to clarify the mechanisms underpinning α-syn transmission along the gut-brain axis, further defining its functional role throughout PD progression.

Mitochondrial dysfunction represents another core mechanism implicated in PD pathogenesis. Impaired mitochondrial function results in inadequate neuronal energy production, elevated oxidative stress, and enhanced neuronal apoptosis, especially pronounced within dopaminergic neurons of patients with PD. Mutations in genes such as PINK1 and parkin disrupt mitochondrial quality control through impaired mitophagy and energy metabolism, thereby accelerating neurodegeneration. Consequently, therapeutic strategies aimed at restoring mitochondrial function, promoting mitophagy, and reducing oxidative stress represent promising avenues for future PD treatment.

Emerging therapeutic strategies targeting the gut-brain axis, including probiotics, anti-inflammatory agents, immunomodulators, and dietary interventions, have shown considerable potential; however, significant challenges remain in their clinical translation. Future PD therapeutic approaches should adopt a precision medicine framework, supported by robust fundamental research and large-scale clinical trials, to facilitate the development of personalized treatment regimens.

This review has systematically discussed the role of gut microbiota in PD pathogenesis, emphasizing the underlying mechanisms mediated via the gut-brain axis and their potential impact on disease progression. PD is a complex neurodegenerative disease with incompletely understood etiology, yet accumulating evidence clearly indicates a critical role for gut microbiota dysbiosis in both disease onset and progression. With advances in translational medicine, the gut microbiota has increasingly been recognized as a key factor in PD pathology. Dysregulation of the gut microbiota not only affects immune responses within the nervous system but also modulates core pathological mechanisms in PD, such as neuroinflammation, pathological aggregation of α-syn, and mitochondrial dysfunction.

Additionally, this review highlights potential therapeutic strategies aimed at modulating gut microbiota, including probiotics, prebiotics, FMT, antibiotics, and dietary interventions. These methodologies exhibit promising therapeutic potential by restoring microbial homeostasis, enhancing intestinal barrier integrity, and regulating immune responses, thereby alleviating PD symptoms and potentially slowing disease progression. Despite encouraging preclinical findings, their clinical application faces numerous challenges, particularly regarding individualized treatment protocols and the standardization of therapeutic efficacy. Therefore, future studies should emphasize precise functional analyses of gut microbiota, detailed mechanistic explorations of the gut-brain axis in PD, and optimization of therapeutic strategies by integrating precision medicine with advanced technologies such as gene editing and nanomedicine.

In conclusion, gut microbiota exert profound influences on the onset and progression of PD. Elucidating the mechanisms underlying the gut-brain axis will provide valuable insights into the early diagnosis, progression monitoring, and innovative therapeutic interventions for PD. Future research should further enhance functional analyses of the gut microbiota, identify beneficial bioactive microbial metabolites, and advance the clinical translation of gut-targeted therapies, thereby opening new avenues for PD treatment.

## Acknowledgments

The schematic figure included in this article was created using BioRender.com. Figure design was completed by Z. Li in 2025 and is available at the following unique citation link: https://BioRender.com/7shvdv3.

This research is supported by the 10.13039/501100003453Natural Science Foundation of Guangdong Province, China (2016A030310384), the Guangzhou Basic Research Program (2024A04J9951), and the National Natural Science Foundation of China (82101341 and 82104625).

## Author contributions

Xiayu Jin and Waijiao Tang contributed to the conception and overall design of the article. Jirui Wei, Xudong Min, and Zhao Yuan performed the literature search and organized relevant references. Zhuolin Du, Zequn Su, and Tianrong Xun contributed to the drafting of sections related to pathophysiology and therapeutic strategies. Qingyao Du, Xiaozheng He, and Waijiao Tang revised the article for intellectual content. All authors reviewed and approved the final version of the article.

## Declaration of interests

The authors declare no competing interests.
